# Clinical and Epidemiological Study of IgA Nephropathy in the Bulgarian Population: Insights into Disease Presentation and Potential Biomarkers

**DOI:** 10.3390/jpm14030269

**Published:** 2024-02-29

**Authors:** Iva Kostadinova, Mila Lyubomirova, Boris Bogov, Ekaterina Kurteva, Dobroslav Kyurkchiev, Todor Todorov

**Affiliations:** 1 Department of Internal Diseases, Medical University of Sofia, 15 “Ivan Geshov” St., 1431 Sofia, Bulgaria; mlyubomirova@medfac.mu-sofia.bg (M.L.); ttodorov@medfac.mu-sofia.bg (T.T.); 2 UMHAT “St. Anna, Clinic of Nephrology”, “Dimityr Mollov” St., 1750 Sofia, Bulgaria; 3 UMAT “St. Ivan Rilski”, Clinic of Immunology, 15 “Ivan Geshov” St., 1000 Sofia, Bulgaria

**Keywords:** IgA nephropathy, biomarkers, Bulgarian population, clinical characteristics, histological features

## Abstract

IgA nephropathy (IgAN) is the most common glomerulonephritis worldwide and a leading cause of chronic kidney disease and renal failure. However, the Bulgarian population has limited epidemiological data and biomarkers for IgAN. In this retrospective monocentric analysis, we investigated all the patients with biopsy-proven IgAN over 10 years in a tertiary Bulgarian institution. From the analysis of 762 kidney biopsies, the diagnosis of primary IgAN was established in 125, with an average age of 35.94 ± 11.91 years. Our study aimed to assess the clinical characteristics, histological features, and potential biomarkers of IgAN in the Bulgarian population. We evaluated parameters such as proteinuria, hematuria, serum creatinine, and glomerular filtration rate (GFR). In fifty IgAN patients and 30 healthy controls, serum levels of Gd-IgA1, IgA, C3, BAFF, and APRIL using ELISA were examined. The results revealed significant differences in serum concentrations of Gd-IgA1 (*p* < 0.001), Gd-IgA1/IgA (*p* = 0.022), IgA (*p* = 0.014), and IgA/C3 (*p* = 0.047) between patients and controls. However, no correlation was found between Gd-IgA1, IgA, Gd-IgA1/IgA, and IgA/C3 and chronic kidney disease progression. Our study reports evidence of the diagnostic value of Gd-IgA1 and contributes to the understanding of IgAN in the Bulgarian population and suggests potential biomarkers for disease diagnosis and prognosis.

## 1. Introduction

IgA nephropathy (IgAN) is recognized as the most prevalent form of glomerulonephritis globally and remains a significant cause of chronic kidney disease and renal failure. This disease exhibits a wide clinical spectrum, ranging from isolated microscopic/macroscopic hematuria and subnephrotic proteinuria to heavy proteinuria and/or declining renal function. Renal biopsy with immunofluorescence is essential for the diagnosis of IgAN. In recent years, research efforts have focused on identifying a biomarker for this disease to aid in diagnosis and monitoring the clinical course [1,2,3,4,5]. The O-linked glycans in the hinge region of IgA1 generally consist of N-acetyl galactosamine (GalNAc) and galactose, with sialic acid potentially attached to either or both sugars. Gd-IgA1 acts as an antigen, combining with autoantibodies to form immune complexes that deposit in the mesangium and initiate downstream action. These immune complexes are nephritogenic, contributing directly to glomerular inflammation and mesangial proliferation. Key mediators of the production of Gd-IgA1 and its corresponding autoantibodies are B-cell activating factor (BAFF) and A proliferation-inducing ligand (APRIL), each playing essential roles in the survival and maintenance of B cells and humoral immunity. Elevated serum levels of both BAFF and APRIL are observed in patients with IgAN and correlate with disease severity [6]. Elevated levels of circulating IgA1 with galactose-deficient, O-linked, hinge-region glycans have been reported in IgA nephropathy patients compared to individuals with non-IgA renal disease and healthy controls in various populations, including Caucasians, African Americans, Japanese, and Chinese [7,8,9,10,11]. Recent studies have found that IgAN has a variable natural progression with 10% to 30% of patients progressing to end-stage renal disease (ESRD) within 10 years of renal biopsy [12]. However, epidemiological studies on the Bulgarian population are scarce, and currently, there is a lack of reliable data regarding serum biomarkers in the Bulgarian population. The latest information, dating back to 1988, indicated a 37% risk of progression to ESRD within 10 years, highlighting the urgent need for updated research in this area [13]. To address this knowledge gap, we conducted a retrospective monocentric study at a Bulgarian tertiary institution to investigate the epidemiology, clinical presentation, and potential biomarkers of IgAN. No data regarding Gd-IgA1 in Bulgarian patients with IgAN have been reported. Therefore, our study aims to evaluate the efficacy of serum Gd-IgA1, IgA, Gd-IgA1/IgA, IgA/C3, C3, BAFF, and APRIL as biomarkers for diagnosing IgAN and determine their correlation with disease severity.

## 2. Materials and Methods

We analyzed renal case records at a tertiary Nephrology Clinic in Bulgaria for 10 years. All patients diagnosed with biopsy-proven IgAN during this period were included in this study. We collected comprehensive medical data for each patient from the time of biopsy and throughout the follow-up period of 1, 3, 5, and 7 years, respectively. Patients with secondary IgAN (*n* = 17) were excluded from the study, which included those with Henoch-Schönlein purpura (*n* = 10), chronic liver disease (*n* = 2), and inflammatory bowel diseases such as Crohn’s disease (*n* = 2) or ulcerative colitis (*n* = 3).

Laboratory investigations were conducted as part of routine glomerular disease evaluation for these patients. We recorded various clinical and laboratory parameters for the remaining 125 patients, including hemoglobin, serum creatinine, glomerular filtration rate (GFR) calculated using the CKD-EPI [14] formula for adults, cholesterol, triglyceride, uric acid, common protein, albumin, proteinuria (24 h urine collection), and spot urine sample analysis. Hypertension was defined as systolic blood pressure ≥140 mm Hg and/or diastolic blood pressure ≥90 mm Hg or treatment with antihypertensive drugs. Anemia was defined by gender-specific criteria of hemoglobin concentrations <120 g/L in males and <110 g/L in females. Hyperuricemia was defined by gender-specific criteria of serum uric acid (UA) >420 μmol/L in males and >360 μmol/L in females. Hypercholesterolemia was defined as a total cholesterol level ≥5.2 mmol/L, and hypertriglyceridemia was defined as a total triglyceride level ≥1.7 mmol/L. Urine protein excretion was calculated from a 24 h urine collection. The primary outcome of this study was chronic kidney disease (CKD) progression, defined as a decline in the eGFR category from the value determined at the time of renal biopsy or doubling of the serum creatinine [14,15]. The kidney biopsies were reclassified according to the Oxford classification of IgAN [16]. 

In addition, we measured serum levels of Gd-IgA1, IgA, complement component C3, B-cell activating factor (BAFF), and a proliferation-inducing ligand (APRIL) using an enzyme-linked immunosorbent assay (ELISA) test kits. A control group of thirty healthy individuals was included for comparison. Serum samples were collected before the day of the biopsy and stored at −80 °C until analysis, with minimal freezing and thawing. The samples were initially diluted 200-fold with the EIA buffer to obtain optical density within the measurement range of the kit (1.56~100 ng/mL). All the samples were tested in duplicates, and the mean values were used for analysis. Ethical approval for the research was obtained from the Ethics Committee, and informed written consent was obtained from all patients and controls. Only participants above 18 years of age were included in the study. The number of participants in each group was determined based on factors such as feasibility, funding availability, and the timing of the biopsy. Descriptive and graphical analyses were performed using IBM SPSS Statistics 25.0, MedCalc Version 19.6.3, and Microsoft Excel 2021. Statistical tests, including Fisher’s exact test, chi-square test, Kolmogorov–Smirnov and Shapiro–Wilk tests, one-way analysis of variance (ANOVA), Student’s *t*-test, Kruskal-Wallis non-parametric test, and multiple binary logistic regression analysis were conducted. A significance level of *p* < 0.05 was applied for all statistical analyses.

## 3. Results

### 3.1. Clinical Characteristics of Patients and Controls

**Figure 1:**
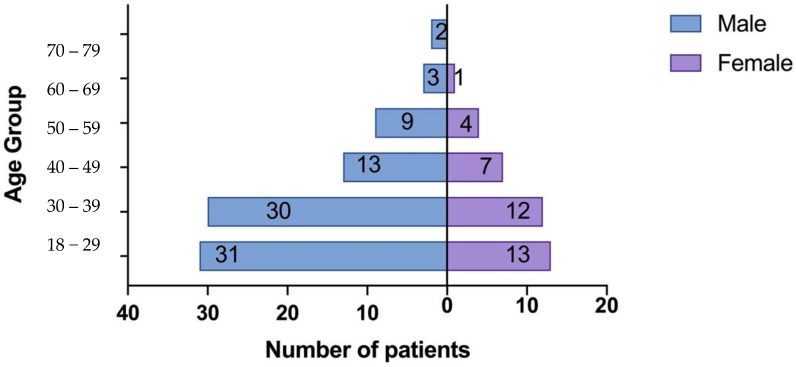
Population Pyramid Frequency age group by sex.

The study included a total of 155 participants, consisting of 125 patients with biopsy-proven IgAN and 30 healthy controls. Among the patients with IgAN, 88 (70.4%) were male, and 37 (29.6%) were female (Figure 1). The mean age of the IgAN patients was 35.94 ± 11.91 years. The baseline characteristics of the patients are summarized in Table 1.

The average baseline estimated glomerular filtration rate (eGFR), calculated using the CKD-EPI formula [14], was 61.78 ± 27.72 mL/min/1.73 m^2^, and the mean range of serum creatinine at the time of kidney biopsy was 126 ± 67.83 μmol/L. Among the patients, 64 (51.2%) had an eGFR below 60 mL/min, indicating CKD stage 3 or worse according to the KDIGO classification [17]. The advanced stage of the disease at diagnosis could be attributed to the long duration from the first symptoms to the kidney biopsy, which was approximately 24.22 ± 32.9 months, as well as the absence of a screening program in Bulgaria. Notably, although 50 patients had experienced episodes of gross hematuria in the past, there was no correlation found between microscopic or macroscopic hematuria and the histological stage of the disease (*p* = 0.714). Hypertension was present in 81 (64.8%) patients, and 37 (29.6%) patients had a history of recurrent throat infections. Only 9 patients (7.2%) underwent tonsillectomy during the follow-up period.

To investigate the factors influencing disease progression based on the glomerular filtration rate (GFR) and doubling of serum creatinine values over time, a binary logistic regression analysis was conducted. The potential factors examined in the study included anemia (hemoglobin < 120 g/L for males and <110 g/L for females), hyperuricemia (uric acid ≥ 420 μmol/L for males and ≥360 μmol/L for females), cholesterol levels ≥ 5.20 mmol/L, and triglyceride levels ≥ 1.7 mmol/L. The analysis revealed that among the tested factors, only anemia showed a significant association with the risk of disease progression based on GFR over time, with a borderline significance level of *p* = 0.053. The risk influence of anemia was approximately 3.4 times Table 2.

Age presented in years; M, male; F, Female; *n*, data presented as numbers; eGFR, estimated glomerular filtration rate; CKD, chronic kidney disease and stage of CKD based on KDIGO, Kidney Disease: Improving Global Outcomes; SD, standard deviation.

When all the examined indicators were included in the regression equation to account for the combined impact and address confounding factors using the “backward conditional” procedure, only anemia remained in the final equation, with its risk influence slightly decreasing to approximately 3.3 times (OR 3.333; CI 0.978–11.365; *p* = 0.054). For the doubling of serum creatinine values, anemia and hyperuricemia were identified as significant factors on an individual level (Table 3).

Anemia had a higher risk influence for disease development (approximately 12 times higher risk), followed by hyperuricemia with approximately 2.4 times higher risk. Including all the examined variables jointly in the regression equation and applying the “backward conditional” procedure, anemia and hyperuricemia remained in the final version of the equation. Their risk influences retained their initial directions and statistical significance, with anemia increasing to around 16 times and hyperuricemia to approximately 3 times.

### 3.2. Histological Characteristics of Patients 

**Figure 2:**
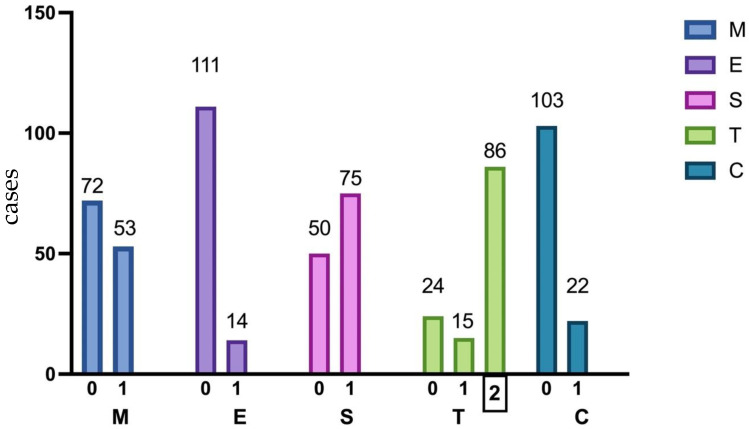
Percentage distribution of pathologic features in 125 kidney biopsies according to the Oxford classification. MEST-C, Oxford classification system; M1, mesangial hypercellularity; E1, endocapillary hypercellularity; S1, segmental glomerulosclerosis; T1/2, tubular atrophy, and interstitial fibrosis >25%; C 1/2, crescent in at least one glomerulus.

Histological analysis based on the Oxford classification [16] of IgAN revealed the presence of mesangial hypercellularity (M1) in 53 patients (42.4%), endocapillary hypercellularity (E1) in 14 patients (11.2%), segmental glomerulosclerosis (S1) in 75 patients (60.0%), and tubular atrophy/interstitial fibrosis (T1/T2) in 101 patients (68.8%) Figure 2.

Twenty-two patients (17.6%) had crescents, among whom 8 presented with rapidly progressive renal failure. The patients were divided into two groups based on the histological severity of IgAN, using the MEST-C score (score 3 or above). No significant correlation was observed between clinical variables such as serum creatinine at the time of diagnosis, hematuria, the duration of complaints, and the MEST-C score Table 4.

Interestingly, a statistically significant relationship was found between the histological stage of the disease based on Haas’s classification [18] and serum creatinine values at diagnosis. Focal-segmental glomerulonephritis showed a statistically significantly higher average value compared to minor histological changes and diffuse proliferative, while it did not differ statistically from focal proliferative Table 5. The analysis did not include patients with the pathohistological result of advanced chronic glomerulonephritis due to a lack of statistical representativeness.

### 3.3. Serum Levels of Key Biomarkers

Serum levels of key biomarkers were assessed in 50 patients and 30 controls. Statistically significant differences were observed between the patient and control groups for four out of the seven measured parameters (Table 6).

**Figure 3:**
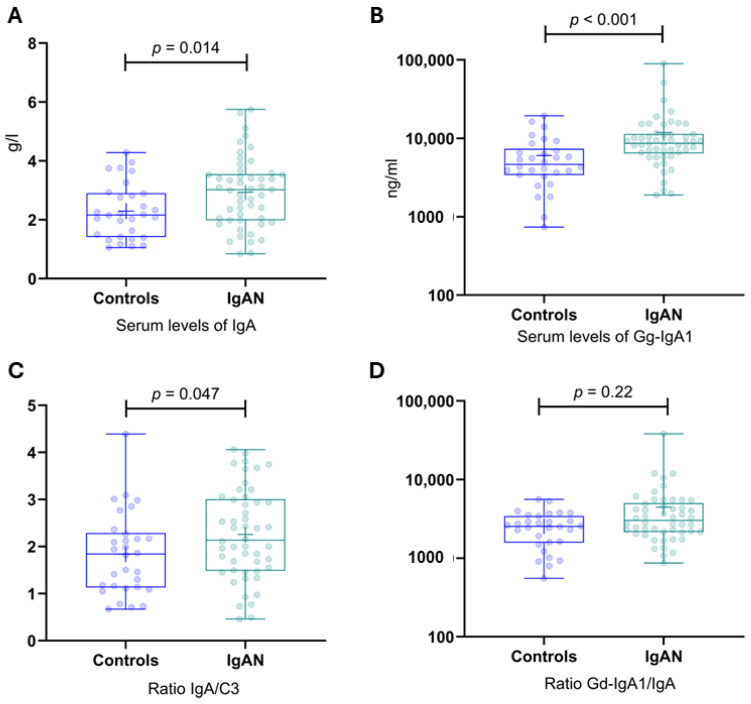
Serum levels of IgA, Gd-IgA1, IgA/C3, and the Gd-IgA1/IgA ratio in IgAN patients and healthy controls. (**A**) Serum levels of IgA in IgAN patients and healthy controls. (**B**) Serum levels of Gd-IgA1 in IgAN patients and healthy controls. (**C**) IgA/C3 ratio in IgAN patients and healthy controls. (**D**) Gal-deficient IgA1/IgA ratio in IgAN patients and healthy controls. Gd-IgA1, galactose-deficient IgA1; IgAN, IgA nephropathy.

Serum concentrations of IgA, Gd-IgA1, IgA/C3, and the Gd-IgA1/IgA ratio were significantly higher in the patient group compared to the control group Figure 3.

**Figure 4:**
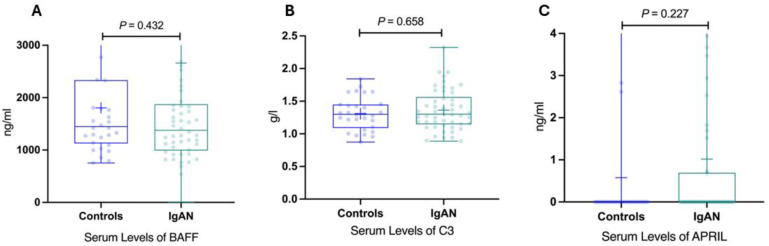
Serum levels of BAFF, C3, APRIL in IgAN patients and healthy controls. (**A**) Serum levels of BAFF in IgAN patients and healthy controls. (**B**) Serum levels of C3 in IgAN patients and healthy controls. (**C**) Serum levels of APRIL in IgAN patients and healthy controls. BAFF, B-cell activating factor; IgAN, immunoglobulin A nephropathy; APRIL, a proliferation-inducing ligand; IgAN, immunoglobulin A nephropathy.

However, no significant differences were noted for C3, BAFF, and APRIL Figure 4.

**Figure 5:**
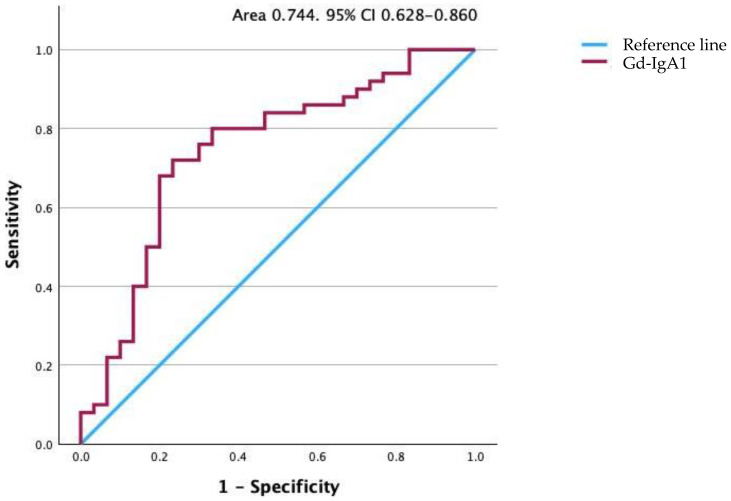
Receiver operating characteristic curve for Gd-IgA1 levels. The area under the curve is 0.747. Gd-IgA1, galactose-deficient IgA1.

The median serum Gd-IgA1 level for the patients of 8435 ng/mL (range 1888–89,642 ng/mL) was significantly higher than the median of 4315 ng/mL (range 738–19,450 ng/mL) for the control group (*p* < 0.001). The serum Gd-IgA level was higher than 5698 ng/mL for 40 of the 50 patients. These data indicate a sensitivity of 80.0% and specificity of 66.7%, with a positive predictive value of 80.0% and a negative predictive value of 66.7%. To illustrate the potential of this assay, the results are also presented as a receiver operating characteristic curve in Figure 5. The area under the curve is 0.744 with a standard error of 0.059, indicating that the true-positive rate was high and that the false-positive rate was low. The 95% confidence interval (CI) was 0.628–0.860 (*p* < 0.001). 

The correlation between clinical features and the levels of serum biomarkers among various subgroups of patients diagnosed with IgA nephropathy are shown in Table 7, providing insights into potential associations and patterns within this patient cohort.

To explore the association between serum Gd-IgA1, IgA, IgA/C3 levels, and CKD progression based on the eGFR, the patients with IgAN were divided into two groups: “CKD progression” included patients with a 25% reduction in eGFR or a decline in the eGFR category from the value determined at the time of renal biopsy, while “CKD non-progression” included the remaining patients who did not meet the criteria for progression. The characteristics of the two groups were compared in Table 8.

No significant correlation was found between serum concentrations of Gd-IgA1, IgA, Gd-IgA1/IgA, and IgA/C3 and the progression of the disease based on GFR over time. This suggests that changes in GFR are not dependent on the levels of Gd-IgA1 and IgA in the serum. Furthermore, Gd-IgA1, IgA, Gd-IgA1/IgA, and IgA/C3 serum concentrations do not appear to be reliable predictors of disease progression based on GFR. In summary, this study suggests that the analyzed parameters, including serum concentrations of Gd-IgA1, IgA, Gd-IgA1/IgA, and the IgA/C3 ratio, may serve as potential diagnostic indicators of the disease. However, their usefulness in predicting disease progression and treatment response is limited. Prospective longitudinal studies are imperative to gain deeper insights into the progression of IgA nephropathy and to authenticate the predictive value of biomarkers. Further research is needed to gain a better understanding of the underlying mechanisms and identify more reliable predictors in this patient population. 

## 4. Discussion

IgA nephropathy (IgAN) is a common primary glomerulopathy known for its slow progression, eventually leading to end-stage renal disease (ESRD) in 30–40% of patients [12,19,20]. Jean Berger described the disease in 1968, and one of his articles, “Worldwide Perspective of IgA Nephropathy,” published in 1988, includes references from Bulgarian authors Belovezhdov et al., dating back to 1984 [21]. This is one of the last articles including data from the Bulgarian population. Another notable study in Bulgaria was conducted in 1988 by Kiperova et al. [13]. These contributions provided valuable insights into IgAN. However, limited data from Bulgarian authors have impeded a comprehensive understanding of the disease in this population. Our study sheds light on the epidemiology, clinical presentation, and potential biomarkers of IgAN among the Bulgarian population for the first time in the last 30 years. In our study population, we observed that 20 patients (17%) developed ESRD (eGFR < 20 mL/min) during the follow-up period, and 23 patients (18.4%) showed established progression of kidney disease based on a doubling of serum creatinine levels. These findings align with the rates reported in the literature. Our cohort’s delayed diagnosis and biopsy, approximately 24.22 months, can be attributed to the absence of a screening program and limited awareness among the Bulgarian population. As a result, 44 patients (35.2%) were diagnosed with the histological variant of IgAN—focal-segmental glomerulosclerosis, likely due to advanced stage at the time of diagnosis and a higher proportion of patients with chronic changes such as interstitial fibrosis and tubular atrophy (19.2%). 

A comparison of our cohort with other study populations in Table 9 reveals similarities and differences. Our study population had a similar age distribution (35.94 years) to the VALIGA [22] cohort (36 years) and slightly higher than the Oxford study cohort [16] (30 years). The gender distribution in our cohort (70.4% males) was also comparable to the VALIGA [22] (73% males) and Oxford (72% males) cohorts. 

The mean proteinuria in our cohort (1.3 g/24 h) was consistent with the VALIGA cohort. However, the Oxford Study [16] and Zeng et al. [24] excluded patients with eGFR less than 30 mL/min, whereas our study included 15 patients (12%) in this category. The distribution of histopathological lesions in our cohort was like the VALIGA [22] cohort, but we observed a higher prevalence of segmental glomerulosclerosis and tubulointerstitial lesions.

It is well known that new biomarkers of IgAN are needed for non-invasive diagnosis and appropriate treatment. The pathogenic events in IgAN are now understood as a four-hit mechanism involving circulating immune complexes (composed of galactose-deficient IgA1) depositing in the glomerulus, leading to mesangial cell proliferation and glomerular injury [25,26]. In our study, we found significantly higher serum concentrations of Gd-IgA1, IgA, Gd-IgA1/IgA, and IgA/C3 in IgAN patients compared to healthy controls. These results are similar to those described in the literature [27,28,29,30,31,32] in other populations but confirmed for the first time in Bulgaria. These findings suggest the potential utility of these biomarkers. However, we observed that these biomarkers were not associated with disease activity or changes in eGFR, indicating limited prognostic utility. Previous studies that examined correlations between serum Gd-IgA1 levels and disease activity and progression have shown diverse results. Moldoveanu et al. [7] confirm that serum levels of Gd-IgA1 differentiated IgAN patients from the healthy controls with high sensitivity, while total levels of serum IgA did not have satisfactory diagnostic value. Our study shows a similar result. Different complement fractions have been shown to have prognostic value in IgAN. Kim et al. [33] reported that decreased serum C3 level (i.e., under 90 mg/dL) predicted poor renal survival, defined as doubling of serum creatinine and renal replacement therapy initiation. This is not confirmed by other studies; moreover, Yang et al. [34] reported that decreased serum C3 levels in IgA nephropathy patients did not play a decisive role in renal progression. Moreover, in several studies from Asia, high serum IgA/C3 ratio—above 3 to 4.5—was a sign of progressive disease, but this has not been confirmed in other ethnic populations [35,36]. Stefan et al. [32] confirm the prognostic value of IgA/C3 ratio in IgAN in Caucasian European patients. Our research does not establish a good prognostic utility of Gd-IgA, IgA, Gd-IgA1/C3, and IgA/C3. 

## 5. Conclusions

Our study offers valuable insights into the clinical and laboratory characteristics of IgA nephropathy in Bulgaria, shedding light on the unique characteristics of this population. Importantly, we have confirmed the diagnostic value of Gd-IgA1 in our population for the first time, highlighting its potential as a reliable biomarker for diagnosing and monitoring IgA nephropathy. Additionally, our study revealed significantly higher serum concentrations of IgA, Gd-IgA1/IgA, and IgA/C3 in the patient group. These findings further emphasize the potential utility of these biomarkers in the accurate diagnosis and effective monitoring of IgA nephropathy. By identifying these associations, we provide a foundation for future research and clinical applications aimed at improving the management and outcomes of patients with IgA nephropathy. One of the main advantages of this study is the extended median follow-up period of approximately 7 years. However, this study has limitations, including its retrospective nature and single-center design, which may introduce selection and referral biases. Future prospective studies involving larger patient cohorts and multiple centers are warranted to validate these findings and enhance our understanding of IgA nephropathy in the Bulgarian population.

Overall, this study sheds light on the clinical and laboratory characteristics of IgA nephropathy in Bulgaria and provides a foundation for future research and clinical management of this prevalent glomerulonephritis.

## Figures and Tables

**Table 1:** Baseline clinical and laboratory profile of patients at the time of biopsy.

	Total Patients (*n* = 125)
Age (yrs) (Mean ± SD)	35.94 ± 11.91
Gender (M:F) (*n*)	88:37
Hb (g/L) (Mean ± SD)	141.37 ± 19.49
Cholesterol (mmol/L) (Mean ± SD)	5.17 ± 1.45
Triglyceride (mmol/L) (Mean ± SD)	1.43 ± 0.78
Serum uric acid (μmol/L) (Mean ± SD)	373.7 ± 112.91
Serum protein (g/L) (Mean ± SD)	69.58 ± 6.69
Serum albumin (g/L) (Mean ± SD)	42.03 ± 5.49
Hematuria microscopic * (*n*)	
+	62
++	12
+++	46
Hematuria macroscopic (episodes)	50
Urinary protein (g/day) (Mean ± SD)	1.34 ± 1.16
Serum creatinine (μmol/L) (Mean ± SD)	125.71 ± 67.83
eGFR (mL/min/1.73 m^2^) (Mean ± SD)	61.78 ± 27.72
CKD stage on the time of biopsy	
1	21
2	40
3a	26
3b	23
4	13
5	2
Timeframe to biopsy	24.22 ± 32.9
Follow up duration	69.60 ± 22.19

*, dipstick sample result.

**Table 2:** Odds ratio and 95% confidence interval of parameters studied as factors for the occurrence of disease progression based on eGFR over the years.

Risk Factors	Range	Individually				Group			
		OR	95% CI		*p* Value	OR	95% CI		*p* Value
			Lower Limit	Upper Limit			Lower Limit	Upper Limit	
Anemia (g/L)	<120 M <110 F	3.352	0.986	11.394	0.053	3.333	0.978	11.365	0.054
Hyperuricemia (μmol/L)	≥420 M ≥360 F	1.023	0.481	2.174	0.953				
Cholesterol (mmol/L)	≥5.2/<5.20	1.313	0.631	2.731	0.466				
Triglyceride (mmol/L)	≥1.7/<1.7	1.094	0.486	2.463	0.829				

OR, Odds ratio; CI, confidence interval. Bold data indicate a significant *p*-value.

**Table 3:** Odds ratio and 95% confidence interval of the studied indicators as factors for the occurrence of disease progression based on a doubling of serum creatinine values.

Risk Factors	Range	Individually				Group			
		OR	95% CI		*p* Value	OR	95% CI		*p* Value
			Lower Limit	Upper Limit			Lower Limit	Upper Limit	
Anemia (g/L)	<120 M <110 F	12.086	3.536	41.306	**<0.001**	15.832	4.203	59.642	**<0.001**
Hyperuricemia (μmol/L)	≥420 M ≥360 F	2.393	0.952	6.016	0.064	3.087	1.031	9.243	**0.044**
Cholesterol (mmol/L)	≥5.2/<5.20	1.036	0.417	2.572	0.939				
Triglyceride (mmol/L)	≥1.7/<1.7	0.680	0.229	2.017	0.486				

OR, Odds ratio; CI, confidence interval. Bold data indicate a significant *p*-value.

**Table 4:** MEST-C correlation with clinical variables: serum creatinine, hematuria, duration of complaints.

Clinical Variables	MEST-C [16]						
	<3			≥3			*p* Value
	*n*	$\bar{X}$	SD	*n*	$\bar{X}$	SD	
**Serum creatinine (μmol/L)**	72	121.63	61.91	53	133.62	75.19	0.560
**Duration of complaints**	68	28.11	36.43	50	18.92	25.84	0.126
**MEST-C**							
	<3			≥3			
Hematuria *	Frequency						0.937
**+**	*n* %	36 50.0		26 49.1			
**++**	*n* %	9 12.5		8 15.1			
**+ + +**	*n* %	27 37.5		19 35.8			
**Episode of macroscopic hematuria**	*n* %	42 58.3		33 62.3			0.714

MEST-C, Oxford classification system [16]; M1, mesangial hypercellularity; E1, endocapillary hypercellularity; S1, segmental glomerulosclerosis; T1/2, tubular atrophy, and interstitial fibrosis >25%; C 1/2, crescent in at least one glomerulus; $\bar{X}$, sample Mean; SD, standard deviation; *, dipstick sample result.

**Table 5:** Correlation between serum creatinine value at diagnosis and histological stage of the disease based on Haas’s classification.

Haas Classification [18]	Serum Creatinine (μmol/L)		
	*n*	$\bar{X}$	SD
Minimal Histological Lesion	15	96.73 ^a^	24.93
FSGS	44	135.09 ^bc^	53.24
Focal Proliferative GN	28	125.71 ^ac^	70.74
Diffuse Proliferative GN	30	117.37 ^a^	69.32
Advanced Chronic GN *	4	271.25	132.46

The same letters indicate the absence of a significant difference, and different letters indicate the presence of one (*p* < 0.05); * this category was not included in the analysis due to a lack of statistical representativeness; $\bar{X}$, sample mean; SD, standard deviation; *n*, number of patients; FSGS, focal segmental glomerular sclerosis; GN, glomerulonephritis.

**Table 6:** Difference between serum concentration of Gd-IgA1, IgA, C3, Gd-IgA1/IgA ratio, IgA/C3 ratio, BAFF, and APRIL in healthy controls and IgAN patients.

Variables	Groups						
	Controls			IgAN Patients			*p* Value
	*n*	$\bar{X}$	SD	*n*	$\bar{X}$	SD	
Gd-IgA1	30	6022.49	4421.79	50	11,882.38	1374.19	**<0.001**
IgA	30	2.29	2.29	50	2.93	1.18	**0.014**
C3	30	1.31	1.31	50	1.36	0.31	0.658
Gd-IgA1/IgA	30	2552.91	2551.91	50	4458.26	5486.84	**0.022**
IgA/C3	30	1.84	0.87	50	2.26	0.97	0.047
BAFF	30	1804.23	956.73	50	2660.40	6925.67	0.432
APRIL	30	0.57	1.67	50	1.017	2.71	0.227

$\bar{X}$, sample mean; SD, standard deviation; Gd-AgA1, galactose-deficient IgA1; IgA, immunoglobulin A; C3; BAFF, B-cell activating factor; APRIL, a proliferation-inducing ligand. Bold data indicate significant *p* values.

**Table 7:** Clinical features and levels of serum biomarkers of patients with IgA nephropathy.

Group	No.	Age	Interval to Kidney Biopsy (Months)	24-h Proteinuria *	Gd-IgA1	IgA	C3	CKD-EPI GFR [17]
	Count	Mean	Mean	Median	Median	Median	Median	Mean
IgAN	50	34.8 ± 9.7	19.4 ± 30.3	1.4 (0.3–5.5)	8637.7 (1888.9–89,642)	3.0 (0.8–5.7)	1.3 (0.9–2.3)	64.4 ± 30.7
Male	33	35 ± 9	20 ± 34	1.3 (0.3–5.5)	7754.25 (980–89,642)	3.0 (1.1–5.7)	1.3 (0.9–2.3)	59.1 ± 28.9
Female	17	34.4 ± 10	16.9 ± 20	0.7 (0.3–3.8)	7030.1 (738.5–30,622.55)	2.8 (0.8–5.6)	1.3 (0.9–1.9)	74.8 ± 32.3
CKD I	10	29.8 ± 7	16 ± 25	1.2 (0.3–5.5)	8930.07 (3676.75–16,376.50)	3.4 (0.9–3.9)	1.3 (1.0–2.3)	109.5 ± 10.9
CKD II	16	32.8 ± 8	18.4 ± 22	0.6 (0.3–3.8)	7954.62 (2140.25–30,622.55)	2.6 (0.8–5.7)	1.3 (0.9–1.7)	76.1 ± 8.7
CKD IIIa	9	40 ± 12	8.6 ± 10	0.7 (0.3–2.6)	9455.95 (1888.95–51,037.7)	3.1 (1.8–5.1)	1.6 (0.9–1.8)	52.8 ± 4.8
CKD IIIb	7	36.1 ± 11	26.7 ± 31	1.1 (0.4–2.8)	8634.15 (3949.4–11,286.45)	3.4 (1.9–4.3)	1.2 (1.0–1.7)	37.2 ± 4.4
CKD IV	7	38.1 ± 10	33.4 ± 60	2.0 (0.5–3.9)	10,965.5 (1970.85–89,642)	2.4 (2.4–3.0)	1.2 (0.9–1.9)	23.0 ± 3.9
ESRD	1	36	6	1.6	15,373.75	4.7	1.4	12.2

CKD, chronic kidney disease; ESRD, end-stage renal disease; GFR, glomerular filtration rate.

**Table 8:** Correlation between serum concentrations of Gd-IgA1, IgA, Gd-IgA1/IgA, and IgA/C3 and disease progression based on eGFR over years.

Variables	CKD Progression Based on eGFR						
	No			Yes			
	*n*	$\bar{X}$	SD	*n*	$\bar{X}$	SD	*p* Value
Gd-IgA1	31	12,353.24	16,685.58	19	11,114.14	6908.02	0.442
IgA	31	2.79	1.23	19	3.16	1.10	0.407
Gd-IgA1/IgA	31	4905.13	6692.2	19	3729.16	2530.77	0.960
IgA/C3	31	2.05	1.00	19	2.58	0.82	0.067

Gd-IgA1, galactose-deficient IgA1; IgA, immunoglobulin A; Gd-IgA1/IgA, galactose-deficient IgA1/immunoglobulin A ratio; IgA/C3, immunoglobulin A/C3 ratio; eGFR, estimated glomerular filtration rate. eGFR was calculated using the CKD-EPI formula [14].

**Table 9:** Comparison of clinic-pathologic characteristics of the present cohort with other study populations.

	Present Study Bulgaria	Oxford [16] Study (Multi-Country)	Valiga [22] Cohort (Europe)	Alamartine et al. [23] (France)	Zeng et al. [24] (China)	Katafuchi et al. [25] (Japan)
Patients (*n*)	125	265	1147	183	1026	702
Age (yrs, mean)	35.94	30	36	38	34	30
Males (%)	70.4	72	73	74,9	50	42
Proteinuria (g/day)	1.34	1.7	1.3	1.2	1.3	0.9
S Cr (μmol/L)	126.7					
eGFR (mL/min/1.73 m^2^)	62	83	73	72	85	82
MEST lesions %						
M1	34.2	80	28	21	43	12
E1	9	42	11	14	11	42
S1	48.4	45	70	54	83	79
T1 and T2 (T2)	55.5 (9.7)		21 (3.6)	30 (10)	27.3 (3.3)	30 (12)
C1	14.2		11	5	48	63
Follow-up duration (months)	69.6	65	56	77	53	62
ACEi/ARB (%)	59.4	74	86	65	89	37
Immunosupression (%)	38.4	29	46	30	31	32
End point definition	Doubling of S Cr	50% decline in eGFR or ESKD	50% decline in eGFR or ESKD	Doubling of S Cr or ESKD	50% decline in eGFR or ESKD	ESKD
END point event (%)	18.4	22	16	20	15.5	12

## Data Availability

Please contact the corresponding author for data access.
